# Development of an automated ultrasonographic detection method for fecal retention using a transgluteal cleft approach

**DOI:** 10.1371/journal.pone.0338926

**Published:** 2025-12-29

**Authors:** Masaru Matsumoto, Yumi Sano, Kazuhiro Akiyama, Katsumi Urata, Natsuki Matsuzaka, Nao Tamai, Yuka Miura, Hiromi Sanada

**Affiliations:** 1 Department of Well-being Nursing, Graduate School of Nursing, Ishikawa Prefectural Nursing University, Kahoku, Ishikawa, Japan; 2 Former Department of Imaging Nursing Science, Graduate School of Medicine, The University of Tokyo, Bunkyo-ku, Tokyo, Japan; 3 Department of Clinical Laboratory, Tokatsu Clinic Hospital, Matsudo, Chiba, Japan; 4 Department of Gastroenterological Surgery, Tokatsu Clinic Hospital, Matsudo, Chiba, Japan; 5 Department of Nursing, Tokatsu Clinic Hospital, Matsudo, Chiba, Japan; 6 Department of Pharmacy, Tokatsu Clinic Hospital, Matsudo, Chiba, Japan; 7 Department of Nursing, Graduate School of Medicine, Yokohama City University, Yokohama, Japan; 8 Former Global Nursing Research Center, Graduate School of Medicine, The University of Tokyo, Bunkyo-ku, Tokyo, Japan; 9 Research Center for Implementation Nursing Science Initiative, Research Promotion Headquarters, Fujita Health University, Toyoake, Japan; 10 Former Department of Gerontological Nursing/Wound Care Management, Graduate School of Medicine, The University of Tokyo, Bunkyo-ku, Tokyo, Japan; 11 Ishikawa Prefectural Nursing University, Kahoku, Ishikawa, Japan; Chattogram Veterinary and Animal Sciences University, BANGLADESH

## Abstract

This study aimed to develop an artificial intelligence–based classification system using ultrasound images obtained via a transgluteal cleft scanning approach for detecting fecal retention in the lower rectum. The goal was to support accurate, objective constipation assessment by nurses in home care settings, where traditional diagnostic tools are often unavailable. Ultrasound videos of the lower rectum were collected from 24 patients undergoing dialysis at a mixed-care hospital. From 90 videos, 2,855 still images were extracted and labeled by expert sonographers based on the presence or absence of hyperechoic areas indicating fecal retention. A deep learning segmentation model using U-Net with a ResNeXt-50 encoder was trained and evaluated. Performance was measured using the intersection over union threshold of 0.5 to define true positives. Accuracy, sensitivity, and specificity were calculated on a test dataset of 758 images. Among the test images, 376 (49.6%) showed fecal retention. The AI system achieved a sensitivity of 81.6%, specificity of 84.0%, and overall accuracy of 82.8%. The mean IoU was 0.601 ± 0.185, indicating a high level of agreement between expert annotations and AI-generated predictions. The tool reliably detected fecal retention in ultrasound images obtained using the transgluteal cleft approach, which overcomes limitations of traditional transabdominal scanning caused by obesity, bladder emptying, or bowel gas. The proposed AI-assisted ultrasound system showed high diagnostic performance in identifying fecal retention in the lower rectum. It may enable non-specialist nurses to assess constipation more safely and accurately, particularly in home-care environments. This technology has the potential to reduce unnecessary laxative use and invasive interventions, ultimately improving the quality of bowel care for older adults with impaired communication or mobility.

## Introduction

Constipation, defined as the infrequent or difficult passage of stools, is one of the most distressing conditions among older adults. It affects approximately 16% of the general adult population and becomes increasingly common with age [1]. In Japan, more than one-third of individuals aged 60 years or older report experiencing constipation, and the prevalence exceeds 50% among nursing home residents [2]. Furthermore, constipation is associated with poor prognoses, including an increased risk of cardiovascular events due to straining during defecation [3,4]. In this context, accurate assessment and timely intervention for constipation in older adults—particularly those receiving home care—are of great importance.

As Japan transitions toward community-based integrated care, home healthcare nurses are increasingly responsible for managing bowel function in aging populations [5]. However, many older adults receiving home care have cognitive or physical impairments, making it difficult to assess subjective symptoms of constipation. Inappropriate administration of laxatives and suppositories without confirming the presence of fecal retention can lead to complications such as diarrhea, mucosal damage, or unnecessary digital disimpaction [6–8]. Therefore, objective assessment methods that allow accurate evaluation of rectal fecal retention are essential to improve the safety and efficacy of bowel care.

Ultrasonography has emerged as a promising tool for the assessment of constipation due to its low cost, safety, portability, and real-time capability [9,10]. Point-of-care ultrasound (POCUS) using handheld devices has become more widely used by nurses in non-radiology settings. In particular, the transabdominal approach using a convex probe enables visualization of rectal fecal retention as a hyperechoic half-moon shape on ultrasound images [11–13]. Educational programs and standardized care algorithms have been developed based on this technique, and its utility in home care has been demonstrated [14].

However, the transabdominal approach is not without limitations. Image acquisition can be hindered by factors such as obesity, insufficient bladder filling, and intestinal gas [15]. To overcome these limitations, a transgluteal cleft approach has been proposed, in which the ultrasound probe is placed between the coccyx and the anus while the patient lies in a lateral decubitus position [16]. This method is less affected by abdominal wall thickness or intrapelvic gas and allows clearer imaging of the lower rectum. It is particularly well-suited for home care settings, where environmental control is more limited than in hospital settings.

Despite its advantages, interpretation of ultrasound images using the transgluteal cleft approach requires specialized skill and experience. To address this, we aimed to develop an automated deep learning-based system to identify rectal fecal retention in handheld ultrasound images. Previous studies have demonstrated the utility of artificial intelligence (AI) in automating ultrasound image interpretation for detecting bladder urine volume, pressure ulcers, vascular structures, and rectal fecal retention using transabdominal approaches [17–21]. However, no study has yet reported an AI-assisted diagnostic tool using the transgluteal cleft approach.

Therefore, the aim of this study was to develop and evaluate a deep learning-based classification system for identifying lower rectal fecal retention using handheld ultrasonography with the transgluteal cleft approach. This AI-powered tool is expected to support non-expert healthcare providers, especially nurses, in accurately assessing constipation in home care settings.

## Materials and methods

### Participants and setting

This study was conducted through a multi-step process consisting of clinical data acquisition, ultrasonographic image interpretation, annotation of relevant features in the images for training purposes, dataset construction (comprising training, validation, and test datasets), and the evaluation of the developed system’s performance. The study site was a regional hospital located in a suburban area near Tokyo, Japan. This facility served as a core center for dialysis treatment and included both acute care and long-term care wards, with a total of 95 beds—60 in acute care and 35 in long-term care.

A multidisciplinary team specializing in bowel care was established at this facility and included a physician, a certified wound, ostomy, and continence (WOC) nurse, a clinical laboratory technician, and a pharmacist. Patients who were evaluated by this team during the study period were eligible for inclusion. All included participants underwent both digital rectal examination and transgluteal ultrasonography to assess fecal retention. These assessments were conducted consecutively to allow direct comparison between clinical and ultrasonographic findings.

Ultrasound imaging was performed between January 2018 and October 2020 by a highly experienced, certified medical sonographer specializing in lower rectal ultrasonography. Ultrasound images were collected at the point of care under routine clinical conditions.

### Data collection

A wireless pocket-sized ultrasound system (iViz wireless; Fujifilm Corporation, Tokyo, Japan) equipped with a convex transducer was utilized for imaging. The ultrasound probe operated at frequencies between 2–5 MHz, with the depth adjusted between 7–10 cm depending on the patient’s body habitus. To maintain hygiene and prevent contamination, the probe was covered with a disposable polyvinylidene chloride sheath during each procedure.

Participants were placed in the left lateral recumbent position. The probe was gently positioned along the gluteal cleft, specifically between the anus and the coccyx, to obtain transverse images of the lower rectum via the transgluteal approach [16]. The ultrasound examinations were recorded as video sequences.

Each video was reviewed by a trained sonographer and classified into two categories based on the presence or absence of hyperechoic signals indicative of fecal retention. From the videos showing stool retention, up to 40 representative frames containing hyperechoic areas were selected. Similarly, from the videos showing no stool, up to 40 frames without hyperechoic areas were extracted.

**Fig 1:**
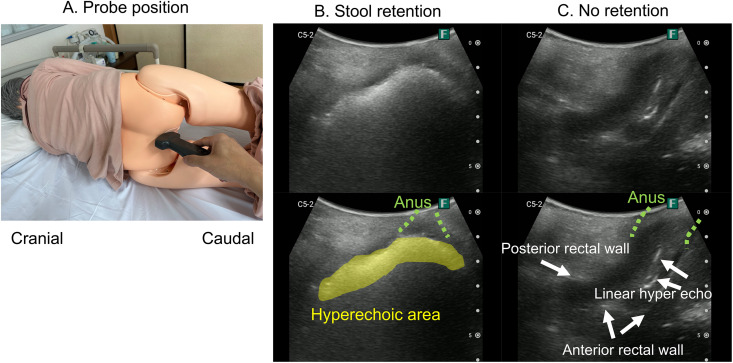
Probe position and rectal ultrasound images in the transgluteal cleft approach scanning method. **(A)** Probe position. A mannequin (or patient) is placed in a left lateral recumbent position with the knees flexed to create a space between the coccyx and the anus. The probe was applied to this area with a longitudinal scan. **(B)** Ultrasound image when there is fecal retention. Feces in the lower rectum are observed as hyperechoic areas. **(C)** Ultrasound image when there is no fecal retention. A hyperechoic line is observed when feces are not accumulated, and even the anterior rectal wall is observed. All photographs and ultrasound frames are the authors’ own; no third-party copyrighted material or logos were reused.

In total, ultrasound video data were collected from 24 patients. Based on a 3:1:2 ratio, 12 patients were allocated to the training dataset, 5 to the validation dataset, and 7 to the test dataset. This yielded a total of 90 ultrasound video clips, distributed as follows: 52 in training, 16 in validation, and 22 in test sets. The videos were also classified into four visual patterns: rock, cotton, mousse, and none (Fig 1). Of the 90 videos, 14 were categorized as rock (15.6%), 31 as cotton (34.4%), 5 as mousse (5.6%), and 54 as none (60.0%).

From these video sequences, 2,855 still images were extracted. Among them, 1,455 images displayed signs of fecal retention, while 1,400 images did not.

### Workflow overview (textual flowchart)

We summarize the end-to-end pipeline below:

1. Data acquisition — Bedside ultrasound was performed via the transgluteal cleft to obtain short video sequences of the lower rectum.
2. Expert review — Experienced examiners identified the image areas that indicate stool retention.
3. Data split — Participants were assigned to training, validation, and test sets at the patient level to avoid information leakage.
4. Model training — A standard segmentation model was trained on the labeled images (full specifications appear in Model development).
5. Evaluation (criterion A: presence/absence) — We judged whether each frame contained stool retention and reported sensitivity, specificity, and accuracy (with 95% confidence intervals).
6. Evaluation (criterion B: overlap-based) — We additionally required sufficient spatial overlap between the model’s region and the expert’s region (details in Evaluation), and reported the same metrics.
7. Error analysis & interpretation — We examined representative mistakes and outlined directions for future clinical validation

### Development of a deep learning-based classification system

#### U-Net.

To develop an automated system for the detection of fecal retention in ultrasound images, we implemented a deep learning-based segmentation approach. Convolutional Neural Networks (CNNs) are widely recognized for their high performance in image recognition tasks such as object detection and semantic segmentation [22].

For this study, we adopted the U-Net architecture, a popular CNN-based model designed for medical image segmentation [23]. The model consists of an encoder for feature extraction and a decoder for generating segmented output maps. We employed a ResNeXt-50 architecture, pretrained on general image datasets, as the encoder due to its balance between model complexity and accuracy [24]. This pretrained encoder was integrated into the U-Net framework to enhance performance under limited data conditions.

#### Training datasets.

**Fig 2:**
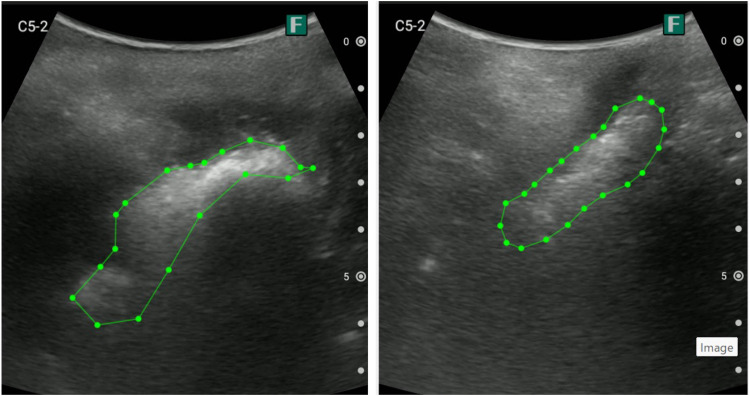
Examples of annotations made by sonographers on ultrasound images. Hyperechoic areas indicating fecal retention in the lower rectum are surrounded by polygons using the annotation tool LabelMe on a PC.

Training of the deep learning model required manually labeled datasets. A nursing researcher selected representative ultrasound images, and a certified sonographer annotated each image by marking regions of hyperechoic signals corresponding to fecal retention in the lower rectum. Negative samples (images without fecal retention) were also labeled accordingly. Fig 2 presents examples of annotation in the training data.

#### Implementation.

The original ultrasound images (800 × 600 pixels) were resized to 512 × 384 pixels while preserving the original aspect ratio as much as possible. This resolution was selected to avoid degradation of visual features during model training and to ensure compatibility with the network’s pooling layers.

To improve model generalization, several data augmentation techniques were applied, including random adjustments to brightness, contrast, and sharpness, as well as horizontal flipping and random erasing. Model training was performed using the Adam optimizer for 100 epochs, with an initial learning rate set at 1 × 10 ⁻ ⁵. All experiments were implemented using Keras and executed on a single NVIDIA GTX 1080 Ti GPU.

### Evaluation of the deep learning-based classification system

All ultrasound images were analyzed using the developed deep learning-based segmentation tool (hereafter referred to as “AI system”). The primary objective was to determine whether a hyperechoic area suggestive of lower-rectal fecal retention was present or absent on each frame.

We evaluated performance under two complementary criteria (see Workflow overview, Step 5–6):

#### A ) Presence/absence criterion (frame-level detection).

Each image was labeled as: (a) presence of a hyperechoic area (fecal retention) or (b) absence of a hyperechoic area (no fecal retention). Under this criterion,

• TP: ground truth (GT) = presence, and the AI predicts any region;
• FN: GT = presence, and the AI predicts no region;
• TN: GT = absence, and the AI predicts no region;
• FP: GT = absence, and the AI predicts any region.

We report sensitivity, specificity, and accuracy computed as:

• Sensitivity = TP/ (TP + FN)
• Specificity = TN/ (TN + FP)
• Accuracy = (TP + TN)/ (TP + TN + FP + FN)

#### B ) IoU-based criterion (segmentation-quality–aware detection).

To assess segmentation quality, we additionally computed the Intersection over Union (IoU) between the AI-predicted region(s) and the expert-annotated region(s):

• IoU = Area of Overlap/ Area of Union.

A frame was counted as positive only if any predicted region achieved IoU ≥ 0.5 with any GT region. Accordingly,

• TP: GT = presence, and IoU ≥ 0.5 for at least one predicted–GT pair;
• FN: GT = presence, and no predicted–GT pair reaches IoU ≥ 0.5 (including IoU < 0.5 or no detection);
• TN: GT = absence, and the AI predicts no region (IoU is not applied to GT-negative frames);
• FP: GT = absence, and the AI predicts any region.

We report sensitivity, specificity, and accuracy under this stricter criterion as well.

When multiple regions exist on either side, the best-matching pair (maximum IoU) is used to determine whether IoU ≥ 0.5 is satisfied for the frame-level decision in criterion B.

### Ethics

This study was approved by the Ethics Committee of the Graduate School of Medicine, The University of Tokyo (approval no. 2020301NI-(1)). Given the sensitive nature of the transgluteal ultrasound procedure, which involves probe placement along the gluteal cleft, special care was taken to respect the privacy and dignity of the participants. Ultrasonographic examinations were performed as part of routine clinical care at the hospital, and ultrasound videos/images were secondarily used for research under an IRB-approved opt-out process; therefore, written informed consent was waived. Study information, including the purpose, methods, and data handling procedures, was made publicly available on the institutional website, and patients—or, when a patient lacked decisional capacity, their family—were able to make an informed choice and decline participation without any disadvantage. All ultrasound data were anonymized at the point of collection and used solely for research purposes. The ultrasound data used for model development were accessed for research purposes on 01/01/2022.

## Results

### Participant characteristics

A total of 24 patients participated in the study. The mean age was 77.2 ± 11.6 years (range, 50–96 years), and 13 participants (54.2%) were female. The average body mass index (BMI) was 19.7 ± 3.4 kg/m². Regarding underlying diseases, 18 patients (75.0%) were undergoing maintenance dialysis, and 11 patients (45.8%) had a diagnosis of diabetes mellitus. One patient (4.2%) had a history of rectal cancer.

### Ultrasonographic dataset composition

From the 24 participants, a total of 90 ultrasonographic videos were acquired. These were allocated into training (n = 52), validation (n = 16), and test (n = 22) datasets. From these videos, 2,855 still images were extracted, consisting of 1,455 images showing fecal retention and 1,400 images without retention. Classification of the stool properties using the transgluteal approach revealed the following distribution: “rock” (n = 14; 15.6%), “cotton” (n = 31; 34.4%), “mousse” (n = 5; 5.6%), and “none” (n = 54; 60.0%), with some overlap or ambiguity in 4 videos (4.4%).

### Performance of the deep learning system

In total, 758 still ultrasound images were used in the final test, of which 376 (49.6%) showed fecal retention and 382 (50.4%) did not show fecal retention in the lower rectum. Table 1 presents the simple accuracy results. The sensitivity and specificity were 93.4% (95%CI: 0.908–0.958) and 84.0%(95%CI: 0.803–0.877), respectively, and the percentage of correct answers was 88.7% (95%CI: 0.863–0.909). The IoU values averaged 0.601 ± 0.185. In the initial presence/absence evaluation, 11 frames (3.1% of all test frames) were counted as positive despite showing no spatial overlap with the expert label. Under the IoU-based criterion (IoU ≥ 0.5), 44 frames initially classified as TP were reclassified as FN; the sensitivity and specificity were 81.6% (95% CI: 0.777–0.855) and 84.0% (95% CI: 0.803–0.877), respectively, and the overall accuracy was 82.8% (95% CI: 0.801–0.855) (Table 2).

**Table 1:** Confusion matrix for frame-level presence/absence of hyperechoic regions (AI vs. expert labels).

		Judgment by the sonographer		
		True	False	Total
Judgment by AI	Positive	351	61	412
	Negative	25	321	346
	Total	376	382	758

**Table 2:** Confusion matrix under an IoU ≥ 0.5 criterion (frame-level detection; AI vs. expert labels).

		Judgment by the sonographer		
		True	False	Total
Judgment by AI	Positive	307	61	368
	Negative	69	321	390
	Total	376	382	758

Presence/absence criterion (frame level). If the ground truth indicates stool is present (hyperechoic region), any predicted region is counted as positive (TP); no predicted region is negative (FN). If the ground truth indicates stool is absent, no predicted region is negative (TN); any predicted region is positive (FP). Abbreviations: AI, artificial intelligence.

IoU-based criterion (frame level). For frames with a ground-truth hyperechoic region (stool present), a prediction is positive (TP) if any predicted region achieves IoU ≥ 0.5 with any ground-truth region; otherwise it is negative (FN), including IoU < 0.5 or no detection. For frames with no ground-truth region (stool absent), no predicted region is negative (TN) and any predicted region is positive (FP). (IoU is not applied to ground-truth–negative frames.) Abbreviations: IoU, intersection over union; AI, artificial intelligence.

**Fig 3:**
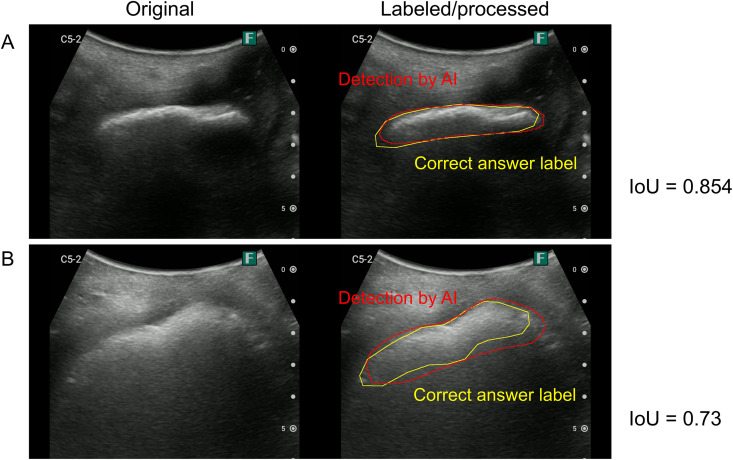
Example of ultrasound image determined as true positive. (Left) Original image. (Right) Areas labeled as correct by the sonographer (yellow) and detected by AI (red). **(A)** IoU = 0.584, **(B)** IoU = 0.73, and IoU > 0.5, resulting in a true positive decision. AI, artificial intelligence; IoU, intersection over union.

**Fig 4:**
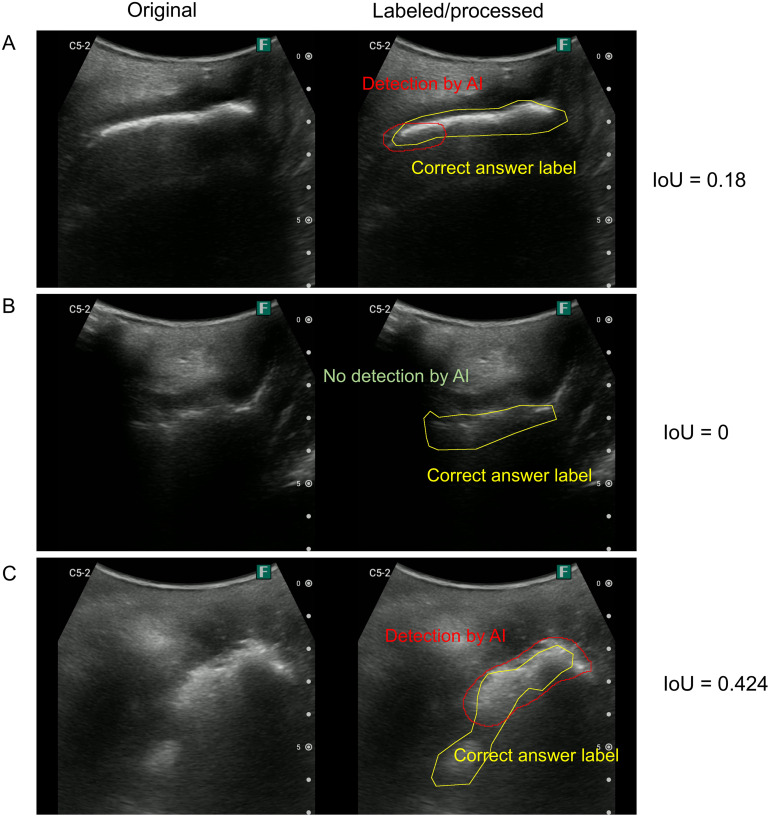
Examples of ultrasound images determined as false negatives. (Left) Original image. (Right) Areas labeled as correct by the sonographer (yellow) and detected by AI (red). **(A)** IoU = 0.18, **(B)** IoU = 0, **(C)** IoU = 0.424, and IoU < 0.5, resulting in a false negative decision. AI, artificial intelligence; IoU, intersection over union.

**Fig 5:**
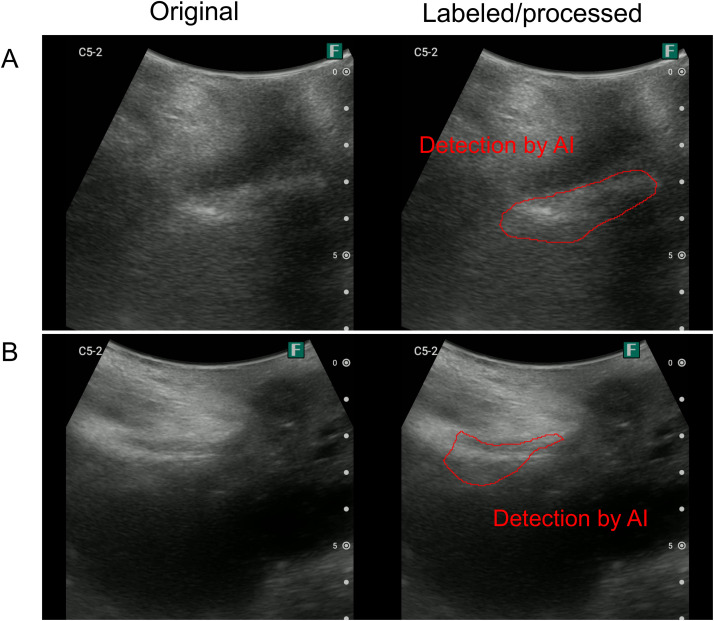
Examples of ultrasound images determined as false positives. (Left) Original image. (Right) Areas labeled as correct by the sonographer (yellow) and detected by AI (red). Despite the sonographer’s determination of no hyperechoic findings indicating fecal retention, the AI misidentified the images as false negatives. AI, artificial intelligence; IoU, intersection over union.

Fig 3 presents an example of ultrasound image of TP because the IoU is > 0.5. Fig 4 and 5 present a typical failure case: the FN image has a pattern in which a mousse-like hyperechoic area is present (suggesting lower rectal fecal retention), a pattern in which the high-echo area is somewhat unclear, and a pattern in which the IoU is < 0.5 but seems to be approximately successful in detecting the stool (Fig 4). Furthermore, in the images that were determined to be FP, there were patterns in which it was difficult to determine whether there was a hyperechoic area and patterns in which there were obvious FPs (Fig 5).

## Discussion

This study demonstrated the technical feasibility of using a deep learning–based segmentation method to detect fecal retention in the lower rectum from sonographer-acquired ultrasonographic images captured via a transgluteal approach using a handheld ultrasound device. The system achieved a sensitivity of 81.6% and specificity of 84.0% (based on IoU ≥ 0.5). Because all images were acquired by professional sonographers, performance on nurse-acquired images remains to be established. The transgluteal ultrasonographic approach has been proposed as a solution to challenges in transabdominal imaging, particularly in home care settings where bladder filling, patient positioning, and gas retention may limit visibility. Our results suggest that the transgluteal method, combined with AI-assisted interpretation, may overcome these limitations by providing clear visualization of fecal matter irrespective of patient obesity or bladder volume.

Although less accurate than previous studies [11,16,18], the automated identification tool developed herein exhibited a high detection accuracy of >80%, indicating its potential for use in clinical observations. We did not perform additional comparative analyses with digital rectal examination (DRE) in this study to avoid scope expansion; however, the prior study has demonstrated the feasibility and diagnostic utility of transgluteal ultrasonography for rectal stool assessment, including stool characterization [16]. We added this citation to contextualize the present findings. The sensitivity and specificity of the AI-based automatic identification tool for rectal US images obtained *via* the transabdominal approach were 100% for the presence or absence of rectal fecal impaction, which is much better than the results of this study [18]. In a previous study by Matsumoto et al., only 42 still images were carefully selected by ultrasonographers for testing, which is very different from the selection method and criteria used in the present study, in which still images were mechanically extracted from video images. The inferior results in this study may be due to the use of still images cut from video images for evaluation. Cutting still images from video images may include some out-of-focus or blurred images. In fact, handheld US devices equipped with an AI-based automatic evaluation tool for transabdominal rectal US images (iViz wireless FWT C5-2 convex probe, Fujifilm, Japan) display AI-based evaluation in real time. Therefore, improving the accuracy of the transgluteal approach using the method employed in this study will be necessary, in which a still image is extracted from a moving image and judged by AI.

To further improve the accuracy of AI, using more images and patterns for machine learning is necessary; furthermore, the criteria for correct answers must be reviewed. Previous studies have reported three patterns of stool images among the transgluteal cleft approaches [16]. The reason why the judgments could not be made correctly for images such as Fig 4A, in which the presence of a hyperechoic area indicating fecal retention can be seen by the naked eye, may be because the three image patterns were not adequately studied. The mousse and unclear groups comprised only 5.6% and 4.4% of the videos used in this study, respectively. Further study with more images from these groups is required. Furthermore, some images in the FNs, such as Fig 4C, could be considered as almost correctly identifying fecal impaction even if the IoU was < 0.5. The accuracy parameter could be further improved by reviewing the criteria for a correct answer, that is, the criteria for defining TP.

The present system was designed and evaluated specifically for detecting fecal retention in the lower rectum using the transgluteal cleft approach. Owing to the anatomical and acoustic characteristics of this acoustic window, sigmoid and more proximal segments were not targeted and were not assessed in this study. As such, the method is intended as a focused, adjunctive tool to rapidly identify or exclude rectal fecal retention and should be interpreted alongside transabdominal ultrasound, bowel symptom history, stool diaries, and clinical examination when a broader assessment of constipation is required. Notably, the transgluteal approach can visualize the lower rectum with high clarity because it interrogates shallower tissues than conventional transabdominal imaging [16], and it can sometimes obviate the need for digital rectal examination when assessing stool retention in the anorectal region [25]. Because appropriate defecation care often depends on whether rectal fecal impaction is present [13,11], nurse education programs [26] together with handheld ultrasound devices and AI-assisted interpretation may help support safer and more consistent care—pending further validation, particularly with nurse-acquired images and in multi-center settings.

One of the strengths of this study lies in its real-world dataset. The image data were collected in an actual clinical setting where defecation management was routinely conducted by a multidisciplinary team, including nurses, physicians, and clinical laboratory technicians. This enhances the ecological validity of our AI model and supports future application in community or home healthcare systems. However, this study has some limitations. Ninety ultrasound videos were used, but the total number of patients was only 24. Because multiple frames were obtained from the same individuals, frame-level metrics may be optimistic owing to within-patient correlation, and the risk of overfitting cannot be fully excluded. Future work should incorporate more videos and greater variability in ultrasound findings. In addition, the dataset was collected at a single site under relatively uniform acquisition conditions, which limits external validity. We also acknowledge that extracting frames from cine loops may yield blurred or out-of-focus images that can lower accuracy. Bedside deployment relies on real-time AI visualization—similar to guide modes on handheld devices—where minor motion and intermittent blur naturally occur. Evaluating performance under these conditions therefore helps quantify the robustness required for practical, point-of-care use. Moreover, this study only used images acquired by sonographers; to examine the effectiveness of the system in actual defecation care by nurses, using images acquired by nurses will be necessary. Finally, although prior studies indicate that characterizing the nature of rectal fecal impaction is feasible [18], the present work intentionally focused on presence versus absence to establish feasibility with a small, clinically acquired dataset. In future work, we will extend to clinically meaningful stool categories (e.g., rock/cotton/mousse/none) using a standardized annotation manual and inter-rater reliability assessment. To improve real-world utility and generalizability, we will prospectively collect nurse-acquired images from multiple centers and home-care settings, ensure balanced samples for each stool category, and evaluate multiclass performance and calibration against clinical outcomes (e.g., need for disimpaction, response to laxatives).

## Conclusion

In this study, we developed a machine learning method to automatically classify fecal retention in the lower rectum using a transgluteal cleft ultrasound scanning approach. This method demonstrated high accuracy in detecting fecal retention in the lower rectum.

## References

[1] Bharucha AE, Pemberton JH, Locke GR 3rd. American gastroenterological association technical review on constipation. Gastroenterology. 2013;144(1):218–38. doi: 10.1053/j.gastro.2012.10.028 23261065 PMC3531555

[2] Bouras EP, Tangalos EG. Chronic constipation in the elderly. Gastroenterol Clin North Am. 2009;38(3):463–80. doi: 10.1016/j.gtc.2009.06.001 19699408

[3] Honkura K, Tomata Y, Sugiyama K, Kaiho Y, Watanabe T, Zhang S, et al. Defecation frequency and cardiovascular disease mortality in Japan: the Ohsaki cohort study. Atherosclerosis. 2016;246:251–6. doi: 10.1016/j.atherosclerosis.2016.01.007 26812003

[4] Sumida K, Molnar MZ, Potukuchi PK, Thomas F, Lu JL, Yamagata K, et al. Constipation and risk of death and cardiovascular events. Atherosclerosis. 2019;281:114–20. doi: 10.1016/j.atherosclerosis.2018.12.021 30658186 PMC6399019

[5] Song P, Tang W. The community-based integrated care system in Japan: Health care and nursing care challenges posed by super-aged society. Biosci Trends. 2019;13(3):279–81. doi: 10.5582/bst.2019.01173 31327797

[6] Darrow CJ, Devito JF. An occurrence of sepsis during inpatient fecal disimpaction. Pediatrics. 2014;133(1):e235-9. doi: 10.1542/peds.2012-2963 24366993

[7] Goodman C, L Davies S, Norton C, Fader M, Morris J, Wells M, et al. Can district nurses and care home staff improve bowel care for older people using a clinical benchmarking tool?. Br J Community Nurs. 2013;18(12):580–7. doi: 10.12968/bjcn.2013.18.12.580 24335790

[8] Toner F, Claros E. Preventing, assessing, and managing constipation in older adults. Nursing. 2012;42(12):32–9; quiz 40. doi: 10.1097/01.NURSE.0000422642.83383.17 23147808

[9] Berger MY, Tabbers MM, Kurver MJ, Boluyt N, Benninga MA. Value of abdominal radiography, colonic transit time, and rectal ultrasound scanning in the diagnosis of idiopathic constipation in children: a systematic review. J Pediatr. 2012;161(1):44-50.e1-2. doi: 10.1016/j.jpeds.2011.12.045 22341242

[10] Perniola G, Shek C, Chong CCW, Chew S, Cartmill J, Dietz HP. Defecation proctography and translabial ultrasound in the investigation of defecatory disorders. Ultrasound Obstet Gynecol. 2008;31(5):567–71. doi: 10.1002/uog.5337 18409183

[11] Tanaka S, Yabunaka K, Matsumoto M, Tamai N, Noguchi H, Yoshida M, et al. Fecal Distribution Changes Using Colorectal Ultrasonography in Older People with Physical and Cognitive Impairment Living in Long-Term Care Facilities: A Longitudinal Observational Study. Healthcare (Basel). 2018;6(2):55. doi: 10.3390/healthcare6020055 29799515 PMC6023545

[12] Matsumoto M, Misawa N, Tsuda M, Manabe N, Kessoku T, Tamai N, et al. Expert Consensus Document: Diagnosis for Chronic Constipation with Faecal Retention in the Rectum Using Ultrasonography. Diagnostics (Basel). 2022;12(2):300. doi: 10.3390/diagnostics12020300 35204390 PMC8871156

[13] Matsumoto M, Yoshida M, Yabunaka K, Nakagami G, Miura Y, Fujimaki S, et al. Safety and efficacy of a defecation care algorithm based on ultrasonographic bowel observation in Japanese home-care settings: a single-case, multiple-baseline study. Geriatr Gerontol Int. 2020;20(3):187–94. doi: 10.1111/ggi.13858 31910312

[14] Kessoku T, Matsumoto M, Misawa N, Tsuda M, Miura Y, Uchida A, et al. Expert Consensus Document: An Algorithm for the Care and Treatment of Patients with Constipation Based on Ultrasonographic Findings in the Rectum. Diagnostics (Basel). 2024;14(14):1510. doi: 10.3390/diagnostics14141510 39061648 PMC11276071

[15] Dahl JJ, Sheth NM. Reverberation clutter from subcutaneous tissue layers: simulation and in vivo demonstrations. Ultrasound Med Biol. 2014;40(4):714–26. doi: 10.1016/j.ultrasmedbio.2013.11.029 24530261 PMC3942094

[16] Sano Y, Matsumoto M, Akiyama K, Urata K, Matsuzaka N, Tamai N. Evaluating accuracy of rectal fecal stool assessment using transgluteal cleft approach ultrasonography. Healthcare. 2024;12(13). 10.3390/healthcare12131251 PMC11241498 38998786

[17] Matsumoto M, Karube M, Nakagami G, Kitamura A, Tamai N, Miura Y, et al. Development of an automatic ultrasound image classification system for pressure injury based on deep learning. Appl Sci. 2021;11(17):7817. doi: 10.3390/app11177817

[18] Matsumoto M, Tsutaoka T, Nakagami G, Tanaka S, Yoshida M, Miura Y, et al. Deep learning-based classification of rectal fecal retention and analysis of fecal properties using ultrasound images in older adult patients. Jpn J Nurs Sci. 2020;17(4):e12340. doi: 10.1111/jjns.12340 32394621

[19] Matsumoto M, Tsutaoka T, Yabunaka K, Handa M, Yoshida M, Nakagami G, et al. Development and evaluation of automated ultrasonographic detection of bladder diameter for estimation of bladder urine volume. PLoS One. 2019;14(9):e0219916. doi: 10.1371/journal.pone.0219916 31487299 PMC6728037

[20] Takahashi T, Nakagami G, Murayama R, Abe M, Matsumoto M, Sanada H. Automated Ultrasonographic Detection of Thrombus and Subcutaneous Edema due to Peripheral Intravenous Catheter. J Association for Vascular Access. 2025;30(2):27–32. doi: 10.2309/java-d-24-00023

[21] Takahashi T, Nakagami G, Murayama R, Abe-Doi M, Matsumoto M, Sanada H. Automatic vein measurement by ultrasonography to prevent peripheral intravenous catheter failure for clinical practice using artificial intelligence: development and evaluation study of an automatic detection method based on deep learning. BMJ Open. 2022;12(5):e051466. doi: 10.1136/bmjopen-2021-051466 35613784 PMC9174762

[22] Russakovsky O, Deng J, Su H, Krause J, Satheesh S, Ma S, et al. ImageNet large scale visual recognition challenge. Int J Comput Vis. 2015;115(3):211–52. doi: 10.1007/s11263-015-0816-y

[23] Ronneberger O, Fischer P, Brox T. U-Net: Convolutional Networks for Biomedical Image Segmentation. In: Medical Image Computing and Computer-Assisted Intervention – MICCAI 2015. Cham: Springer INnternational Publising; 2015.

[24] Xie S, Girshick R, Dollar P, Tu Z, He K. Aggregated residual transformations for deep neural networks. 2017;:5987–95.

[25] Japan Academy of Nursing Science. Clinical practice guidelines for colonic fecal retention assessment during constipation for nursing care. 2023.

[26] Matsumoto M, Yoshida M, Miura Y, Sato N, Okawa Y, Yamada M, et al. Feasibility of the constipation point-of-care ultrasound educational program in observing fecal retention in the colorectum: A descriptive study. Jpn J Nurs Sci. 2021;18(1):e12385. doi: 10.1111/jjns.12385 33174689

